# The First Step Awry

**Published:** 1858-12

**Authors:** 


### THE FIRST STEP AWRY.

MANY years since, while traveling with a young friend, through an Indian wilderness, the road diverged very slightly, but seemed to make only a little island, and we felt sure, that we would meet again a short distance ahead. He took the left. I kept the main path. First moments, then miles passed, and dark night came on, rainy and cold and drear enough, but we met no more! Thus it is in the world of mind. A man sets out in the vigorous pursuit of truth, all fresh with youth and hope and energy. But he comes to a point in his progress where the road separates, ever so slightly it may be, seeming more apparent than real, and relying on the seeming, rather than the sure, the wrong path is taken. A false conclusion is adopted, every succeeding step is but a farther divergence from the right, until truth is lost sight of, the mists of error hang thick on every side, and that mind goes out in the darkness of delusions, worse than death.

Precisely so in the moral world. The conscience, so tender of wrong, when its possessor first breaks away from a blessed mother's sight and voice and influence, starts almost at its own soft, sweet voice. But as the day of life wears on, it loses its extreme sensitiveness, and while the young man "would not for the world," do what was positively forbidden, circumstances sometimes occur, where action is required without an express "thus saith the law," or a decided forbiddance; or if there be the slightest misgiving of a probability of wrong, there is a treacherous feeling that "it will all come right again," by prompt action, if circumstances require it; or that it will be rectified before it can be noticed. But "the first step awry" gives direction to a second still more diverging, till in the course of weeks or months, or weary years, the man is waked up to the startling truth, that he is at a returnless distance from home and heaven.

In the pursuits of literature, there are similar resemblances. The young at first read books of a standard character in science and in morals. Then come temptations to swerve from this straight path, and equivocal volumes are gingerly handled; next, those which they would not exactly care to be seen perusing. By degrees the tastes are vitiated, and they return no more to safe and solid reading, but go farther and farther away from purity and truth, and they feed on the trashy monthly, the Sunday newspaper, the low love-storied weekly; and going further down, revel in "yellow covered literature," the infidel magazine, the ribald novel; and last of all, books on "physiology" and "marriage," fill up the measure of moral corruption and ultimate bodily disease.

In the simple habits and practices of youthful life, we grow up to tastes and appetites healthful and safe; but once turning aside to indulgences, irregularities, stimulations, and pamperings, before he is aware of it, the unconscious victim has lost his relish for all that is simple, and safe, and pure, and good; and tobacco and wine, and seasoned food, and unbridled lust for mere animal gratification, disease the body, impair the intellect, debase the heart, and to simplicity of life and purity of taste, and their almost infallible attendant, glorious, joyous health, there is a return, never!

In mind, in morals, and in medication, let all beware of "the first step awry." Its beginning is in doubt. Its progress is in misgiving first, then, infatuation; its end, the irretrievable ruin of intellect, and heart, and body; for all, together, go down in the night of mental error, of moral degradation, and of physical death. Hence we repeat with an emphasis, BEWARE of "THE FIRST STEP AWRY."

---

*Ranking's Abstract*, semi-annual, two dollars a year, LINDSAY & BLAKISTON, Philadelphia—an English reprint, containing the gist of medical progress throughout the world, for six months—should be on the table of every intelligent physician.

